# Growth and longevity modulation through larval environment mediate immunosenescence and immune strategy of *Tenebrio molitor*

**DOI:** 10.1186/s12979-023-00409-w

**Published:** 2024-01-12

**Authors:** Agathe Crosland, Thierry Rigaud, Charlène Develay, Yannick Moret

**Affiliations:** Biogéosciences (UMR-CNRS 6282), Université de Bourgogne, Dijon, France

**Keywords:** Aged immune response, Invertebrate immunity, Disposable soma theory, Constitutive and inducible immunity, Mealworm beetle

## Abstract

**Background:**

The Disposable Soma Theory of aging suggests a trade-off between energy allocation for growth, reproduction and somatic maintenance, including immunity. While trade-offs between reproduction and immunity are well documented, those involving growth remain under-explored. Rapid growth might deplete resources, reducing investment in maintenance, potentially leading to earlier or faster senescence and a shorter lifespan. However, rapid growth could limit exposure to parasitism before reaching adulthood, decreasing immunity needs. The insect immunity’s components (cellular, enzymatic, and antibacterial) vary in cost, effectiveness, and duration. Despite overall immunity decline (immunosenescence), its components seem to age differently. We hypothesize that investment in these immune components is adjusted based on the resource cost of growth, longevity, and the associated risk of parasitism.

**Results:**

We tested this hypothesis using the mealworm beetle, *Tenebrio molitor* as our experimental subject. By manipulating the larval environment, including three different temperatures and three relative humidity levels, we achieved a wide range of growth durations and longevities. Our main focus was on the relationship between growth duration, longevity, and specific immune components: hemocyte count, phenoloxidase activity, and antibacterial activity. We measured these immune parameters both before and after exposing the individuals to a standard bacterial immune challenge, enabling us to assess immune responses. These measurements were taken in both young and older adult beetles. Upon altering growth duration and longevity by modifying larval temperature, we observed a more pronounced investment in cellular and antibacterial defenses among individuals with slow growth and extended lifespans. Intriguingly, slower-growing and long-lived beetles exhibited reduced enzymatic activity. Similar results were found when manipulating larval growth duration and adult longevity through variations in relative humidity, with a particular focus on antibacterial activity.

**Conclusion:**

The impact of growth manipulation on immune senescence varies by the specific immune parameter under consideration. Yet, in slow-growing *T. molitor*, a clear decline in cellular and antibacterial immune responses with age was observed. This decline can be linked to their initially stronger immune response in early life. Furthermore, our study suggests an immune strategy favoring enhanced antibacterial activity among slow-growing and long-lived *T. molitor* individuals.

**Supplementary Information:**

The online version contains supplementary material available at 10.1186/s12979-023-00409-w.

## Background

Aging, also known as senescence, is the gradual deterioration of physiological function and heightened susceptibility to age-related diseases and mortality. It is a phenomenon that impacts nearly all living organisms [1]. Various theories have been proposed to elucidate the proximate mechanisms of aging, such as cell senescence, telomere shortening, oxidative stress, and mitochondrial dysfunction, among others [2–4]. However, these theories do not provide a comprehensive explanation for the ultimate mechanism driving the evolution of aging and its diverse manifestations among different species and within populations [1, 5].

Evolutionary theories of aging are founded on the fundamental premise that the strength of natural selection declines as individuals age since they have already made successful contributions to their lifetime reproductive success [6–9]. Thus, the accumulation of mutations with late-life harmful effects [7] and genes with negative pleiotropic effects—beneficial early in life but then deleterious later in life—might be selected for [9]. The relationship between age-specific selection pressure and aging is often linked to the concept of trade-offs [10], which is central to the disposable soma theory of aging [11], providing a physiological explanation for the aforementioned antagonistic pleiotropy theory [12, 13]. As individuals age, they face trade-offs in allocating limited resources between preserving their own bodies through somatic maintenance and investing in reproduction [11]. During early life, there is a strong selection for investing in rapid growth and reproduction to ensure the successful transmission of genes to the next generation. Consequently, this emphasis on allocating resources for early growth and reproduction may lead to a trade-off with later somatic protection, including immunity, ultimately resulting in a potentially shorter lifespan.

Immunity plays a crucial role in somatic protection. The immune system is a complex network of cells, tissues, and molecules that work together to defend the body against harmful pathogens or cancerous cells [14, 15]. As individuals age, the immune system’s efficiency may decline, resulting in immune senescence, a phenomenon marked by reduced responsiveness to infections and heightened vulnerability to age-related diseases. One potential cause of immune senescence is that, while highly beneficial, immunity is a resource-demanding function that can be subject to trade-offs with growth and reproduction. Numerous observational and experimental studies have examined the relationships between reproduction, adult longevity, and immunity, yielding contrasting results, depending on the species’ reproductive tactics aimed at maximizing fitness [1, 16–18]. Conversely, research specifically investigating the relationship between growth, adult longevity, and immunity is relatively scarce and primarily limited to observational studies [19]. Considering that rapid growth is believed to reduce somatic defenses, it is logical to anticipate that accelerated growth could affect adult immunity and lead to more pronounced immunosenescence. On the other hand, slow growth, coupled with a corresponding increase in adult longevity, is likely to elevate the susceptibility to extrinsic mortality due to prolonged exposure to pathogens. As a result, it is expected that a relatively robust allocation to immune defenses will be maintained as adults age to enhance overall fitness.

Here we aim to study the relationships between growth, adult longevity and age-specific immunity using the mealworm beetle, *Tenebrio molitor*, for which we have experimentally modified growth duration and adult longevity. *T. molitor* is a pest of stored grain products that recently attracted attention as a promising alternative protein source for food and feed purposes [20, 21]. The larval development period of this species is highly variable, ranging from two to six months, and can be influenced by the quality and quantity of nutrition and environmental conditions, such as temperature and relative humidity. As variations in food intake may influence numerous physiological functions beyond simply affecting the trade-off between growth, longevity, and immunity [22], we decided to manipulate the larval environment. To achieve this, we exposed the larvae to three different temperature and three relative humidity conditions.

In *T. molitor*, like in many insects, the immune system relies on both constitutive and inducible innate mechanisms, which encompass cellular and humoral components [23]. Cellular immunity involves four types of hemocytes, namely granulocytes, plasmatocytes, prohemocytes and oenocytoids, responsible for functions such as phagocytosis, nodulation, and encapsulation of endogenous organisms [23–27]. The innate immune response also involves crucial humoral enzymatic defenses, including the activation of the prophenoloxidase (proPO) system [23, 28]. The enzymes of this system are synthesized constitutively and present in the haemolymph, oenocytoids and granulocytes [27]. The activation of the proPO into phenoloxidase (PO) enzyme is central to the inflammatory response [25, 29], leading to the production of cytotoxic compounds [30–33]. These compounds can result in self-damage and ultimately reduce the individual’s lifespan [34–39]. Additionally, microbial infections trigger the synthesis and release of antimicrobial peptides into the hemolymph [40–44]. These peptides may persist for several days, serving either to target microbial pathogens evading immune cells and PO activity [45]. They can also prepare the insect for subsequent pathogenic attacks through immune priming [46], a phenomenon observed in insects and other invertebrates where their immune system exhibits enhanced responses upon re-exposure to a previously encountered pathogen [47]. Remarkably, antimicrobial peptides exhibit relative specificity and are efficient in eradicating microbes without harming the host’s tissues [48]. These different components of the *T. molitor* immune system were found to exhibited contrasting patterns of variation with age [49, 50], suggesting they might incur different age-specific costs. Cellular and enzymatic defenses tend to have lower upfront developmental costs but higher operating costs with a late-life impact that could shorten lifespan [37, 50]. On the other hand, the synthesis of antibacterial peptides appears to be high early in life, and once triggered, the cost of maintenance in later life may remain relatively modest [49].

Therefore, we predict that insects exhibiting rapid growth and short adult longevity, as a result of experimental manipulation of their larval environment, should not heavily invest in costly immune components that primarily act over the long term, such as antibacterial activity and, to a lesser extent, hemocytes. Instead, they may prioritize the enzymatic component of immunity, such as the proPO system.

## Results

### Growth duration, pupal mass and adult survival under different temperature and relative humidity conditions at the larval stage

Larvae were maintained at three different temperatures until pupation (20.5 ± 1 °C, 24 ± 1 °C, and 28.5 ± 1 °C) with relative humidity (RH) of 85 ± 5%, designated as T20, T24 and T28 respectively. Additionally, three relative humidity conditions were applied (55 ± 5%, 70 ± 5%, and 85 ± 5% RH) at a constant temperature of 24 ± 1 °C, designated as H55, H70 and H85 respectively. Notably, the T24/H85 condition was common to both types of environmental modification. Subsequently, they were transferred in a common garden at 24 ± 1 °C and 70 ± 5% RH.

**Fig. 1:**
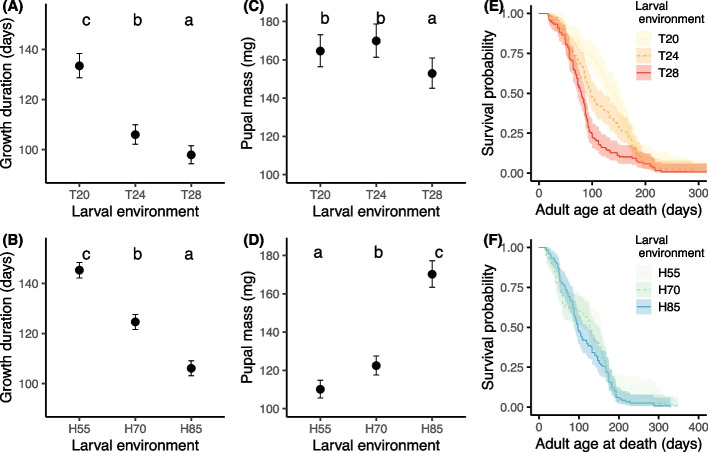
Life history traits after exposure to different larval conditions. **A**, **B** Growth duration (estimated marginal means) as a function of larval temperature (RH constant at 85%) (**A**) and larval relative humidity (temperature constant at 24 °C) conditions (**B**), corrected for the effect of sex. **C**, **D** Pupal mass (estimated marginal means) as a function of larval temperature (**C**) and larval relative humidity (**D**). **E**, **F** adult age specific survival as a function of larval temperature (**E**) and larval relative humidity conditions (**F**). Samples sizes are: T20: 55 females and 63 males; T28: 74 females and 45 males; T24/H85: 51 females and 66 males; H70: 68 females and 52 males; H55: 57 females and 56 males. Error bars and colorful outlines surrounding survival curves are 95% confidence intervals. Different letters represent values significantly different (*p* ≤ 0.05) after Bonferroni-corrected pairwise comparisons of estimated marginal means

Both variations in temperature and humidity of larval rearing had a significant impact on the duration of larval growth (Temperature: LR = 229.5; d.f. = 2; *p* < 0.001, Relative Humidity: *F* = 238.2; d.f. = 2; *p* < 0.001; Figs. 1A and B). Among larvae reared at different temperatures, those raised at 28.5 °C exhibited the fastest growth (Fig. 1A), reaching the pupal stage approximately 8 days ahead of the larvae grown at 24 °C and 36 days earlier than the larvae grown at 20.5 °C (Fig. 1A). The latter had the longest larval growth duration. Among larvae grown at different relative humidities (Fig. 1B), those grown at 85% RH showed faster growth than those grown at 70% RH, while the growth of larvae reared at 55% RH was the slowest among the groups.

Among the groups of insects exposed to different temperature conditions at the larval stage, females grew significantly faster than males (LR = 5.18; d.f. = 1; *P* = 0.023). The same trend was found among insects exposed to different relative humidity conditions, although the difference was marginal (*F* = 3.26; d.f. = 1; *P* = 0.072).

Both temperature and relative humidity during the larval stage had a significant impact on pupal mass (LR = 12.6; d.f. = 2; *P* = 0.002 and LR = 362.0; d.f. = 2; *P* < 0.001 respectively) (Fig. 1C and D). Among the larvae reared at different temperatures, those grown at 20.5 or 24 °C exhibited similar pupal masses. In contrast, the pupae grown at these two temperatures were heavier than those grown at 28.5 °C (Fig. 1C), which also reached this stage faster (Fig. 1A). Concerning larvae reared at different relative humidities, the pattern of pupal mass was opposite to that growth duration (Fig. 1B and D). Pupae from larvae grown at 55%RH were lighter, while those from larvae grown at 85% RH were heavier compared to the two other humidity conditions. Sex did not have a significant effect on pupal mass under the different temperature or relative humidity conditions (LR = 3.76; d.f. = 1; *P* = 0.052 and LR = 1.01; d.f. = 1; *P* = 0.31 respectively).

The temperature variations at the larval stage had a significant impact on adult survival, with higher temperatures lowering adult survival (Fig. 1E and Table 1A). On the other hand, the relative humidity of the larval stage had no significant effect on adult survival (Fig. 1F and Table 1B). While males and females from different larval temperatures survived similarly (Table 1A), adult males survived longer than females when exposed to varied larval relative humidity conditions (Table 1B).

**Table 1:** Cox regression model analyzing adult longevity as a function of larval temperature and sex (A), and larval relative humidity and sex (B). ß is the regression coefficient of the overall survival function for variables, while s.e. is the standard error of the regression coefficient. Odds indicates the odds ratio of survival for variable (exp(b)) relative to the control factor as the reference category. The same model was calculated with T24 and T28 as reference categories to obtain all relative odds ratios. For Sex, females were the reference category. Wald is the statistical test for the variable, and p indicates the significant level for Wald statistic. The sample size for (A) are as follows: T20—55 females and 63 males; T24—51 females and 66 males; T28—74 females and 44 males. The sample size for (B) are as follows: H85—51 females and 66 males; H70—68 females and 52 males; H55—57 females and 56 males

(A)							
Variables	ß	s.e	Odds	Wald	χ^2^	d.f	*P*
Temperature					25.5	2	** < 0.001**
T20 vs T24	-0.28	0.13	0.75	-2.15			**0.03**
T28 vs T24	0.39	0.14	1.48	2.92			**0.004**
T20 vs T28	-0.68	0.13	0.51	-5.04			** < 0.001**
Male vs Female	-0.11	0.11	0.89	-1.031	1.06	1	0.303
(B)							
Variables	ß	s.e	Odds	Wald	Chisq	d.f	*P*
Relative humidity					3.73	2	0.16
H55 vs H70	-0.13	0.13	0.88	-0.97			0.33
H85 vs H70	0.13	0.13	1.14	0.99			0.33
H55 vs H85	-0.26	0.13	0.77	-1.93			0.053
Male vs Female	-0.28	0.11	0.76	-2.55	6.52	1	**0.011**

### Principal component analyses to summarize immune measures

Since only a few oenocytoids were observed in the hemolymph (out of 1004 individual samples counted, only 376 contained at least one oenocytoid, and among these, a mean of two oenocytoids was counted), oenocytoid count could not be analyzed statistically. They were nevertheless included in the total concentration of hemocytes.

We conducted Principal Component Analyses (PCAs) on the measured immune parameters (granulocytes, plasmatocytes, total hemocytes, PO, total-PO, and antibacterial activity) in order to condense them into composite variables. The distribution of the immune parameters across the first three axes are the same than reported in [49]. These first three components of the analyses accounted for 82.2% and 82.1% of the variability for temperature modification and relative humidity modification, respectively (Table 2). The first component mainly represents cellular defenses, including the total concentration of hemocytes and the count of the different hemocyte types (Table 2). The second component predominantly represented proPO defenses as evidenced by the strong loadings of PO activity and total-PO activity (Table 2). Lastly, the third component primarily corresponded to antibacterial activity (Table 2).

**Table 2:** Principal Component Analysis (PCA) loadings of variables and variability of principal component (PC) for larval temperature and relative humidity. Only individuals with complete immune measurements were included (T20: *n* = 88; T28: *n* = 85; T24/H85; *n* = 97; H55: *n* = 92; H70: *n* = 86). The variable loadings associated with each axis are indicated in bold

	Temperature			Relative humidity		
	PC1 (42.7%)	PC2 (24.1%)	PC3 (15.5%)	PC1 (42.4%)	PC2 (24.3%)	PC3 (15.4%)
Total hemocytes	**0.55**	0.14	-0.16	**0.56**	0.10	-0.16
Granulocytes	**0.47**	0.09	-0.36	**0.46**	0.08	-0.41
Plasmatocytes	**0.49**	0.15	0.04	**0.51**	0.09	0.07
Prohemocytes	**0.38**	0.14	0.25	**0.37**	0.17	0.22
PO activity	-0.21	**0.67**	-0.06	-0.16	**0.69**	-0.03
Total-PO activity	-0.19	**0.67**	-0.12	-0.17	**0.67**	-0.17
Antibacterial activity	0.13	0.15	**0.87**	0.15	0.16	**0.85**

The three components of *T. molitor* immunity (cellular defenses, proPO defenses, and antibacterial activity) were estimated using the coordinates derived from the three primary components obtained from the aforementioned PCA. For each type of immunity and specific larval environmental modification, mixed models were formulated, incorporating random effects for larval environmental condition and individuals, given that two measurements were taken per insect (before and after the immune challenge). In addition, these random effects were integrated into the most comprehensive model, which included the following variables and their interactions: growth duration or adult longevity (both in days), immune challenge (before vs. after the immune challenge), and the insect’s age when hemolymph samples and immune challenges were made (referred to as ‘Age’: young vs. old). In order to account for individual variation in quality, pupal mass was also included in all models, without interacting with other variables. A comprehensive model selection process, based on AIC criteria was, carried out for each model. Additional details regarding the best model selections can be found in the Additional file 1.

### Influence of growth duration and longevity on cellular defenses (PC1) through larval temperature modification

**Fig. 2:**
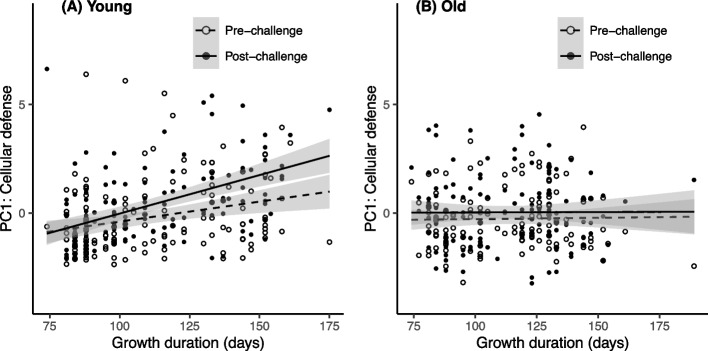
Cellular defense scores (PC1 of the PCA) in insects reared under different larval temperatures. Immune scores are plotted against growth duration, before and after an immune challenge in **A** young adult insects (15 ± 2 days post eclosion) and **B** old adult insects (45 ± 2 days post eclosion). The lines represent linear regression according to growth duration and 95% confidence intervals are depicted in grey

Cellular defense scores (PC1) were generally stronger in heavier adult individuals, and showed, as anticipated, an increase after the immune challenge (β = 0.012 ± 0.005, Table 3A, B). Of greater significance, these cellular defense scores were significantly influenced by the triple interaction involving growth duration, age at measurement and immune challenge (Table 3A). In particular, young insects exhibited an overall elevation of cellular defenses alongside larval growth duration, with this effect amplified after the immune challenge in individuals with extended growth durations (Fig. 2A). Conversely, such effects were absent in older individuals (Fig. 2B). Thus, insects with longer larval stages demonstrated an inclination to allocate increased resources towards cellular defenses during their early adulthood compared to their older counterparts.

**Table 3:** Generalized linear mixed models describing the effects of variables on the three defense scores of adults *T. molitor* that experiences larval grown at different temperature (T20, T24 and T28). The presented models are the most comprehensive ones minimizing the AIC criteria, including either (A) Growth duration or (B) Adult longevity, along with Immune challenge (before vs. after the immune challenge), Age at measurement (hemolymph sampled in young vs. old insects), their two-way and three-way interactions. Body mass of the insects measured before the immune challenge and sex were also included as main effect only. The larval temperature conditions and individual identity were included as random effects. The sample sizes for each category are as follows: T20—40 individuals measured young and 48 old; T24/H85—48 individuals measured young and 37 old; T28—50 individuals measured young and 47 old

Variables	PC1			PC2			PC3		
	Wald χ^2^	d.f	*P*	Wald χ^2^	d.f	*P*	Wald χ^2^	d.f	*P*
(A) Models selected with growth duration as variable in the complete model									
Growth	0.016	1	0.9	0.57	1	0.45	6.76	1	**0.009**
Challenge	16.1	1	** < 0.001**	53.1	1	** < 0.001**	1161	1	** < 0.001**
Age	1.48	1	0.22	-	-	-	14.9	1	** < 0.001**
Mass	8.8	1	**0.003**	2.88	1	0.09	-	-	-
Sex	-	-	-	-	-	-	-	-	-
Growth*Challenge	3.08	1	0.08	5.29	1	**0.02**	-	-	-
Growth*Age	-	-	-	-	-	-	-	-	-
Challenge*Age	-	-	-	-	-	-	8.99	1	**0.003**
Growth*Challenge*Age	20.4	2	** < 0.001**	-	-	-	22.6	3	** < 0.001**
(B) Models selected with adult longevity as variable in the complete model									
Longevity	1.73	1	0.19	4.55	1	**0.03**	5.01	1	**0.03**
Challenge	16.2	1	** < 0.001**	55.1	1	** < 0.001**	1116	1	** < 0.001**
Age	1.88	1	0.17	-	-	-	17.0	1	** < 0.001**
Mass	20.2	1	** < 0.001**	4.31	1	**0.04**	-	-	-
Sex	-	-	-	-	-	-	-	-	-
Longevity*Challenge	-	-	-	3.13	1	0.077	-	-	-
Longevity*Age	-	-	-	-	-	-	-	-	-
Challenge*Age	1.14	1	0.29	-	-	-	8.62	1	**0.003**
Longevity*Challenge*Age	-	-	-	-	-	-	6.76	3	0.08

In contrast to growth duration, adult longevity did not influence cellular defense scores, either as a main effect or in interaction with age at measurement and the immune challenge (Table 3B).

### Influence of growth duration and longevity on proPO defenses (PC2) through larval temperature modification

As anticipated, the immune challenge led to an increase in proPO defense scores (Table 3A, B). In contrast to cellular defense scores, heavier insects exhibited lower levels of proPO defense scores, although this was only statistically significant in the model assessing the influence of adult longevity (β = -0.004 ± 0.003, Table 3B).

**Fig. 3:**
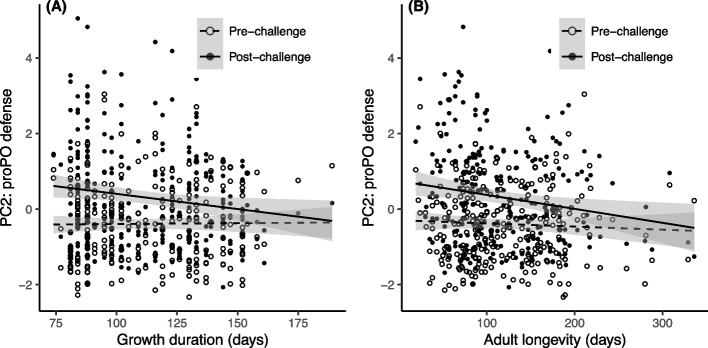
Enzymatic defense scores (PC2 of the PCA) in insects reared under different larval temperatures. Immune scores are plotted against **A** Growth duration and **B** Adult longevity, before and after an immune challenge. Lines represent linear regressions and 95% confidence intervals are depicted in grey

The magnitude of the enzymatic response to the immune challenge was significantly influenced by growth duration (Table 3A). Specifically, individuals with faster growth exhibited a stronger response (i.e. higher levels of immunity after challenge) compared to those with slower growth (Fig. 3A). A similar trend was observed in relation to adult longevity, where short-live insects demonstrated a stronger enzymatic response compared to longer-live ones (Fig. 3B), even though the interaction between adult longevity and immune challenge was only marginally significant (Table 4B). However, the overall significant negative correlation observed between levels of proPO defense scores and adult longevity (Table 3B, Fig. 3B) indicates that long-lived insects allocated fewer resources to proPO defenses compared to short-lived ones.

**Table 4:** Generalized linear mixed models describing the effects of variables on the three defense scores of adults *T. molitor* that experiences larval grown at relative humidity (H55, H70 and H85). The presented models are the most comprehensive ones minimizing the AIC criteria, including either (A) Growth duration or (B) Adult longevity, along with Immune challenge (before vs. after the immune challenge), Age at measurement (hemolymph sampled in young vs. old insects), their two-way and three-way interactions. Body mass of the insects measured before the immune challenge and sex were also included as main effect only. The larval humidity conditions and individual identity were included as random effects. The sample sizes for each category are as follows: H85—48 individuals measured young and 37 older; H70—35 individuals measured young and 41 older; H55—44 individuals measured young and 47 older (one outlier for growth duration was removed for all analysis)

Variables	PC1			PC2			PC3		
	Wald χ^2^	d.f	*P*	Wald χ^2^	d.f	*P*	Wald χ^2^	d.f	*P*
(A) Models selected with growth duration as variable in the complete model									
Growth	0.16	1	0.69	2.89	1	0.09	1.17	1	0.28
Challenge	21.5	1	** < 0.001**	42.9	1	** < 0.001**	781	1	** < 0.001**
Age	-	-	-	1.72	1	0.19	19.3	1	** < 0.001**
Mass	6.78	1	**0.009**	-	-	-	-	-	-
Sex	3.58	1	0.058	-	-	-	-	-	-
Growth*Challenge	-	-	-	-	-	-	-	-	-
Growth*Age	0.19	1	0.66	-	-	-	1.51	1	0.22
Challenge*Age	-	-	-	3.62	1	0.06	-	-	-
Growth*Challenge*Age	-	-	-	-	-	-	6.24	2	**0.04**
(B) Models selected with adult longevity as variable in the complete model									
Longevity	0.81	1	0.37	-	-	-	6.18	1	**0.013**
Challenge	22.8	1	** < 0.001**	44.8	1	** < 0.001**	749	1	** < 0.001**
Age	-	-	-	1.16	1	0.28	23.8	1	** < 0.001**
Mass	6.31	1	**0.012**	-	-	-	-	-	-
Sex	4.20	1	**0.04**	-	-	-	-	-	-
Longevity*Challenge	2.35	1	0.13	-	-	-	-	-	-
Longevity*Age	-	-	-	-	-	-	-	-	-
Challenge*Age	-	-	-	3.72	1	0.054	1.89	1	0.17
Longevity*Challenge*Age	-	-	-	-	-	-	9.33	3	**0.025**

### Influence of growth duration and longevity on antibacterial defense (PC3) through larval temperature modification

**Fig. 4:**
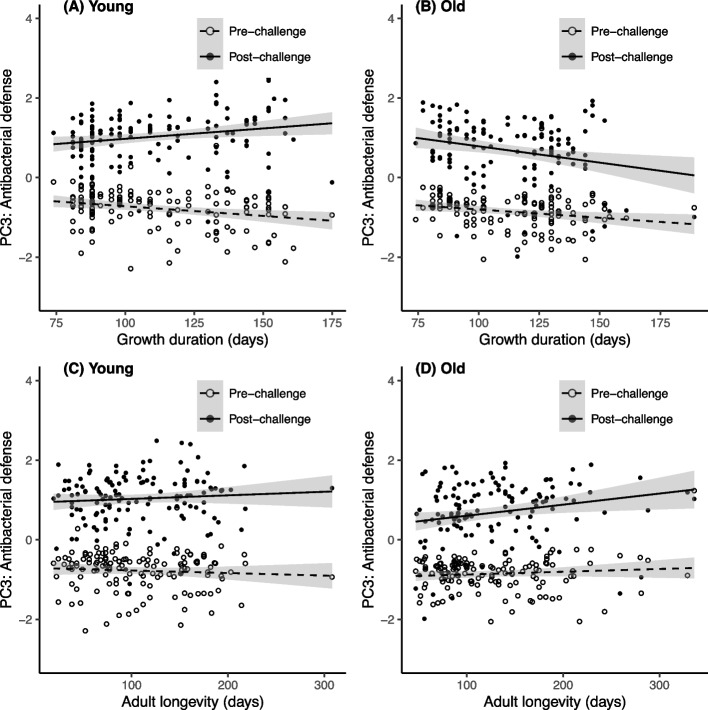
Antibacterial defense scores (PC3 of the PCA) in insects reared under different larval temperatures. Immune scores are plotted against **A**, **B** growth duration and **C**, **D** adult longevity, before and after an immune challenge occurring when the insects were young (**A**, **C**) and when the insects were old (**B**, **D**). The lines represent linear regressions, and 95% confidence intervals are depicted in grey

The antibacterial defense scores exhibited a substantial overall increase in response to the immune challenge (Table 3A, B; Fig. 4). However, the significant interaction between the immune challenge and age at measurement indicates that the magnitude of this antibacterial response is weaker in aged beetles compared to young ones (Table 3A, B; Fig. 4). This age-related shift in the antibacterial response was significantly modulated by growth duration, as indicated by the significant triple interaction (Table 3A). Specifically, among young insects, long growth durations were associated with a stronger antibacterial response to the immune challenge compared to individuals with shorter growth duration (Fig. 4A). On the contrary, among older insects, longer growth durations were associated with a lower antibacterial response to the immune challenge compared to those with shorter growth durations (Fig. 4B).

While adult longevity exhibited an overall positive correlation with antibacterial scores (β = 0.002 ± 0.001, Table 3B, Fig. 4C, D), its triple interaction with the immune challenge and age at measurement was only marginally significant (Table 3B). Specifically, adult longevity showed no significant effect on the antibacterial response to the immune challenge when the response was measured when insects were young (Fig. 4C), whereas long-lived individuals tended to show a stronger antibacterial response when measured in older insects (Fig. 4D).

### Influence of growth duration and longevity on cellular defenses (PC1) through larval relative humidity modification

Although growth duration and adult longevity were kept in the models, neither had a significant effect on cellular defense scores when the larval environment was modified through changes in the relative humidity (Table 4). As expected, the immune challenge had a positive effect on the cellular response, resulting in an average increase of β = 0.6 ± 0.13 of the cellular defense score after the immune challenge. Additionally, consistent with the results obtained with the temperature changes during the larval stage, insects with higher body mass exhibited higher cellular defense scores compared to lighter ones (β = 0.008 ± 0.004, Table 4). In contrast to prior results, the sex of the individuals influenced the levels of cellular defense scores. Males exhibited slightly higher cellular defense scores than females, as shown by the marginally significant effect in the model involving growth duration and the significant effect in the model involving adult longevity (Table 4). According to the latter model, males had an increase of β = 0.34 ± 0.17 in cellular defense scores.

### Influence of growth duration and longevity on proPO defenses (PC2) through larval relative humidity modification

**Fig. 5:**
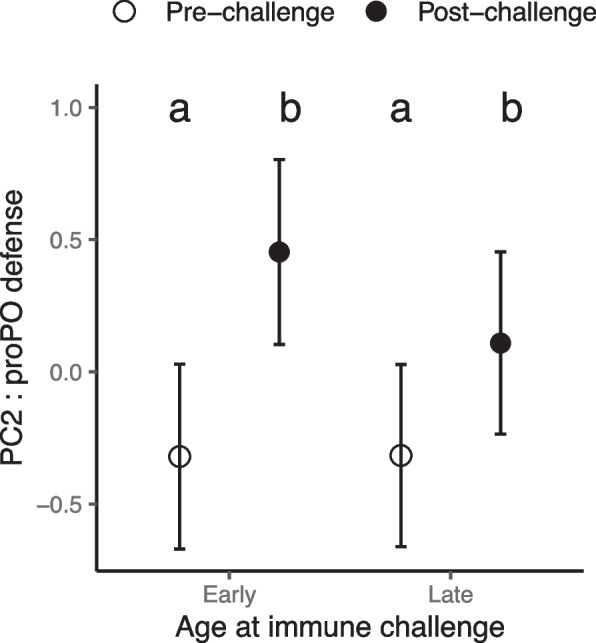
Enzymatic defense scores (PC2 of the PCA) in insects reared under different larval relative humidities. Estimated marginal means are given before (circles) and after (triangles) an immune challenge, occurring either when young or old. Error bars are the 95% confidence intervals. Different letters represent values significantly different (*p* ≤ 0.05) after Bonferroni-corrected pairwise comparisons of estimated marginal means

Neither growth duration nor adult longevity had a significant effect on proPO defense scores, although growth duration was retained in the model (Table 4). The immune challenge significantly increased the enzymatic response. This effect was only slightly influenced by the age of the measurement (Table 4A, Fig. 5). Specifically, the enzymatic response tended to be slightly weaker in older insects (Fig. 5).

### Influence of growth duration and longevity on antibacterial defense (PC3) through larval relative humidity modification

**Fig. 6:**
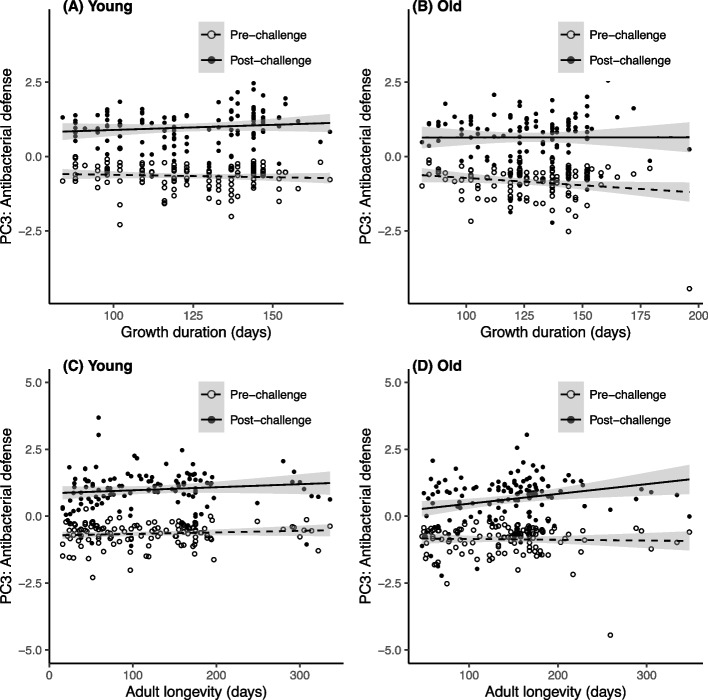
Antibacterial defense scores (PC3 of the PCA) in insects reared under different larval relative humidities. Immune scores are plotted against **A**, **B** growth duration and **C**, **D** adult longevity, before and after an immune challenge occurring when the insects were young (**A**, **C**) and when the insects were old (**B**, **D**). The lines represent linear regressions and 95% confidence intervals are depicted in grey

As previously observed for other immune components, the immune challenge resulted in an increase in the levels of antibacterial defense scores (Fig. 6). However, this effect of the immune challenge was modulated by both the age of the immune challenge and the growth duration (Table 4A, Fig. 6A and B), along with the adult longevity (Table 4B, Fig. 6C and D). These two variables had distinct effects. Specifically, while the antibacterial response appeared to be constant along growth duration among young insects (Fig. 6A), it displayed a slight increase for individuals with longer growth durations in later life (Fig. 6B).

The impact of adult longevity was similar, but more pronounced: when measured in older individuals, the antibacterial response was significantly higher in long-lived adults compared to short-lived ones (Fig. 6D); however, this distinction was not apparent in measurements taken from young insects (Fig. 6C). Similar to the results from the larval temperature modification, it seems that this pattern was primarily driven by a weaker response in older short-lived individuals, rather than a substantial amplification of the response in older long-lived adults in contrast to young adults (Fig. 6C and D).

## Discussion

According to the Disposable Soma Theory of Aging [11], growth and reproduction are assumed resource-demanding functions, leading to trade-offs with somatic maintenance and defense, including the immune system, resulting in a gradual decline of all organismal functions with age. As a result, rapid growth is anticipated to compromise the expression of immune defenses during adulthood, contributing to reduced adult survival. Conversely, slow growth, coupled with an associated increase in adult longevity, is expected to raise the risk of extrinsic mortality due to prolonged exposure to pathogens. Consequently, it is expected that a relatively strong investment in immune defenses will be maintained as adults age to optimize fitness.

Our study investigated how the changes in growth duration and adult longevity, resulting from manipulations of environmental conditions during larval stages, influence adult age-specific investment in innate immune defenses of *T. molitor*. Consistent with previous work [51–53], we successfully manipulated both growth duration and adult longevity of our insects by experimentally exposing them to different temperature or relative humidity conditions during the larval stage. However, the magnitude of the effects of larval temperature and larval RH on growth duration and adult longevity was slightly different. Variation in larval temperature significantly impacted both growth duration and adult longevity, whereas variation in larval RH affected growth duration without impacting adult longevity significantly. Among the larvae reared under different relative humidity conditions, those maintained at 85%RH during their larval stage were the fastest to reach adulthood. Given that they were also heavier than the individuals from other relative humidity conditions, we proposed that the absence of evidence for the expected trade-off between growth and longevity might be attributed to a ‘silver spoon effect’ [54–56]. This effect arises when individuals experience favorable conditions during the initial stages of their life, potentially enabling them to evade the trade-off that typically accompanies rapid growth, namely, a shorter adult life and reduced investment in immunity. A similar phenomenon was observed in a previous study, although in that case, the silver-spoon effect did not mask the heightened reproductive senescence associated with the acceleration in growth rate [51]. Overall, growth duration increased when larval temperature or larval RH decreased, and adult longevity increased when larval temperature decreased. By manipulating the larval environment, we generated substantial range of variations in growth duration and adult longevity. This variation provided us with the opportunity to evaluate their respective effects on the different components of adult immunity in response to a bacterial immune challenge as individuals aged. Of course, since some environmental conditions used here are deviating from the optimal conditions for *T. molitor*, we acknowledge that extreme values may represent stress for this insect, even if it was moderate since no mortality was observed during the larval exposure to the different conditions used here. Nevertheless, these conditions might affect the immune system directly. It is known, for example, that temperature variation directly affects the immune system functioning in various invertebrates [57, 58]. However, these effects are often complex and not directly scaled with environmental conditions [59, 60]. Here, the different environmental conditions are taken into account is our analyses and the effect of growth duration or adult longevity are found, even after controlling for the confounding effects of these direct environmental influences.

### Cellular defenses were modulated by growth duration and age

We found measurable variation in cellular defenses according to age, the immune challenge, and growth duration. As observed in previous studies, including different insect species [49, 61, 62], cellular defenses were observed to decline with age, and in response to the immune challenge, insects circulated more immune cells in the hemocoel [63–65]. However, this heightened response was observed solely among young adult insects and was positively linked to their growth duration. Specifically, only young adults that experienced a longer growth period were capable of sustaining a high baseline concentration of immune cells and, in addition, exhibited a significant increase in their circulation following the immune challenge.

No relationship between cellular defenses and adult longevity was found in our study, suggesting that it was growth rather than adult longevity that mainly drove the variation in this immune component. Furthermore, high investment in cellular defenses was strongly associated with high adult body mass, an indicator of body condition [66], suggesting that cellular defenses are potentially costly and requires good body condition to be maintained. A possible interpretation of these results is that cellular defenses required significant physiological resources and time for its development and the payoff of investing in this arm of the immune system is likely in the long term.

### proPO defenses were modulated by growth duration but not by ageing

ProPO defenses was responsive to the immune challenge, leading to an increase in its activity, but was uncorrelated with age. This latter result is consistent with previous findings showing no significant change or only a weak increase in the activity of the proPO system with age in adult *T. molitor* and *Tribolium castaneum* [49, 50, 67]. The proPO system is an important constitutive immune component in insects, and its activity requires fine-tuned regulation because PO activity produces potentially toxic reactive nitrogen and oxygen species, which can harm the host [30, 38]. Consistently, in *T. molitor*, the stimulation of the PO response was found to reduce longevity in adult beetles [37, 39, 50], and the strength of the response has been found to be negatively correlated with the remaining lifespan [36]. Consequently, investment in this enzymatic component of the innate immune system is expected to pay off in the short term only and may therefore benefit primarily fast-growing insects with short adult lifespans. Consistent with this, we found that the magnitude of the enzymatic immune response to the immune challenge was modulated by growth duration and marginally by adult longevity. Specifically, the enzymatic immune response was stronger among adults that grew faster and had shorter lifespans compared to adult insects that grew slower and had longer lifespans.

It is also noteworthy that, in contrast to cellular defenses, heavy adult insects, which were presumably in good condition and found previously to exhibit longer life span [51], tended to exhibit lower enzymatic immune scores than lighter ones. It therefore appears that investment between constitutive cellular and proPO defenses might be strategic to maximize fitness depending of growth duration and adult life span.

### Inducible antibacterial defense is driven by strong interactions between ageing and growth duration or adult longevity

Antibacterial defense was strongly increased by the immune challenge, as expected for this inducible component of the insect immune system [48]. However, the magnitude of the response decreased with age, possibly indicative of immune senescence, where older individuals are less able to respond to the immune challenge than younger ones. It is noticeable that the contrary was found in *Drosophila melanogaster* [68] and *T. castaneum* [50] with increased antibacterial activity in older individuals.

The antimicrobial immune response involves the inducible production of antimicrobial peptides, and in contrast to the constitutive enzymatic immune response, it is relatively specific in its action and does not induce self-harm. However, this specificity comes at the cost of slow responsiveness [29]. In *T. molitor* larvae, this immune pathway plays a key role in the phenomenon of immune priming, wherein antibacterial activity can be sustained for several weeks after a primary bacterial challenge, offering protection against subsequent infections [46]. Since immune priming is anticipated to provide long-term benefits, investment in antibacterial defense in *T. molitor* is of paramount importance, particularly among slow growers and long-lived individuals who are likely to encounter prolonged exposure to repeated pathogen attacks.

We observed that both growth duration and adult longevity influenced the age-specific expression of the antibacterial immune response during the adult stage of *T. molitor*. However, the effects of growth duration and adult longevity were somewhat divergent. Overall, growth duration showed a negative correlation with antibacterial defense. Nevertheless, long growth duration tended to enhance the antibacterial immune response among young insects, while it was associated with a weaker response among older insects. On the other hand, adult longevity exhibited a positive correlation with antibacterial defense and improved the antibacterial immune response among older individuals. Therefore, it appears that, unlike cellular defenses, antibacterial defense was primarily influenced by adult longevity rather than growth duration, where the latter seemed to incur some costs in old adults that grew slowly.

The positive correlation between adult longevity and the antibacterial response supports our hypothesis, suggesting that maintaining this relatively low-cost immunity might prevent reinfections in long-lived individuals. However, the unexpected finding was the relatively negative impact of larval growth duration. Specifically, despite the fact that young slow-growing insects could develop robust antibacterial immune responses, they demonstrated weaker antibacterial responses as they aged. This trend of variability was evident among both insects raised under fluctuating temperatures and those reared in varying relative humidity conditions, although the impact was more subtle in the latter group. This accelerated decline of the antibacterial immune pathway in slow-growing insects could not be attributed to a potential size-related constraint on their ability to generate antibacterial responses in later stages of life, as it seems to occur among birds [69]. On one hand, as mentioned earlier, slow-growing insects seemed to display the highest responsiveness early in their lives. On the other hand, while extended growth periods resulted in larger adults for insects reared under varying larval temperatures, the same prolonged growth duration was linked to smaller adults among insects raised under fluctuating relative humidity levels. A plausible explanation is that the impact of the growth cost on the antibacterial immune response is likely not linear. While rapid growth might incur costs on immunity, it is also possible that an excessively extended growth duration could be constraining, leading to an accelerated senescence of the inducible antibacterial response. Nevertheless, it should be noted that such a negative impact of an excessively extended growth duration on the antibacterial response late in life might have limited effects on fitness for two main reasons. First, as observed earlier, prolonged growth is associated with robust immune responses early in life when the residual reproductive value (i.e., the potential for future reproduction) of the insects is still high. Second, in the later stages of life, the residual reproductive value of the insects diminishes, and the necessity for a strong capacity to respond to pathogen attacks becomes less critical for individual fitness. This might, therefore, provide an explanation for why accelerated senescence among slow-growing insects could be selected.

We did not analyze the effect of reproduction here, which can trade-off with somatic maintenance or growth rate, nor did we explore the impact of the changes evidenced here on offspring quality and/or on trans-generational immune priming [70]. In *T. molitor*, complex patterns of relationships have been identified between changes in immune defenses with age and female reproduction. While cellular and proPO defenses showed some patterns of ageing and did not tradeoff with reproduction, antibacterial immune response were less affected by age, but highly connected to female fecundity [50]. Comparing the influence of growth on these trade-off patterns would therefore be an exciting and promising prospect.

## Conclusions

We have demonstrated that the pattern immune senescence following growth and longevity manipulation depends of the specific immune parameter under consideration. Limited evidence of aging was observed for enzymatic activity, whereas slow-growing *T. molitor* exhibited an age-related decline in their cellular and antibacterial immune responses. This entailed a robust immune response early in life, transitioning to a weaker or non-existent response in older individuals. This trend was primarily observed in the context of antibacterial activity, an immune component that displayed a negative correlation with growth duration (although a strong response was evident in young adults of slow-growing individuals) but a positive correlation with adult longevity (with a more pronounced response in long-lived older adults compared to younger ones). This implies that an immune strategy favoring antibacterial activity in slow-growing and long-lived individuals has been favored in *T. molitor*. However, the manifestation of senescence in this immune component was linked specifically to slow larval growth. The maintenance of elevated levels of antibacterial response in older adults with the highest probability of survival underscores the significance of this immune response in mitigating the risk of reinfection associated with an extended lifespan.

## Methods

### Insect cultures and experimental design

Age-controlled experimental larvae were obtained from the progeny of insects in an outbred stock culture maintained at the university of Bourgogne (Dijon, France). A total of 150 females and 100 males, 14 to 21 days old post-eclosion, were kept together for 3 days in a plastic tank measuring L 56.5 × l 36.5 × h 15 cm. The tank was filled with bran flour and maintained under standard laboratory conditions, including 24 ± 1 °C, 70 ± 5% relative humidity and continuous darkness. When the larvae reached 4 weeks of age (measuring between 0.8 to 1.2 cm in size, which allowed us to manipulate them without damage (preventing uncontrolled mortality before adulthood), 600 of them were randomly selected, isolated in grid boxes (boxes with 10 compartments each compartment: L 4.6 × l 3 × h 3 cm) and supplied ad libitum with a 9:1 mix of bran flour:fish food (JLB, Novo Malawi). The grid boxes were then haphazardly distributed among five temperature and relative humidity (RH) experimental conditions. Three of these experimental conditions varied in RH: 55 ± 5%, 70 ± 5%, and 85 ± 5%, while the temperature was kept constant at 24 ± 1 °C. The purpose of setting these environmental conditions was to affect larval development by experimentally varying the relative humidity while keeping the temperature constant. These RH values frame the optimal range of 60–75% (as reviewed in [20]). Additionally, we aimed to modify larval development by varying the environmental temperature while keeping the relative humidity constant. For this purpose, we established two additional environmental conditions with temperature variations: 20.5 ± 1 °C and 28 ± 1 °C, while maintaining the relative humidity at 85 ± 5%, leading to three different temperature conditions (20.5 ± 1 °C, 24 ± 1 °C, and 28 ± 1 °C). These temperature values surrounded the optimal range estimated at 23–28 °C [20]. While all our environment manipulations can be stressful for animals at their extreme values, they were far from being deleterious (we observed no mortality during the larval growth). In all these environmental conditions, larvae were kept under continuous darkness.

Survival and development of the larvae were monitored twice a week until pupation. The growth duration was calculated from the date of egg-laying (determined as the median of the 3-day period during which the parents reproduced) to the date of pupation. After pupation, each individual was transferred in a new grid box compartment and maintained in a common garden at the standard laboratory condition (24 ± 1 °C, 70 ± 5% RH, and continuous darkness). Pupae were observed twice a week until eclosion. Newly emerged adults were weighted using an OHAUS Discovery DV114C balance (d = 0.1 mg) and their sex was determined. Adults were maintained under standard laboratory conditions and provided with the same food mixture as during their larval stage, along with a weekly supply of a small apple cube (approximately 4 mm in size) as a source of sugar and water. The survival of adults was assessed weekly until the death of all individuals.

Adults were randomly assigned to age-specific measurements of their immunity either early in their life (around 16 ± 2 days post-eclosion, referred as young individuals) or later (around 45 ± 2 days post-eclosion referred as old individuals). For both age groups, we evaluated individual immunity by conducting hemolymph samplings before and 3 days after an immune challenge to test for the concentration of hemocytes, the antibacterial activity and the maintenance and use of the prophenoloxidase system. These measurements were taken under unchallenged conditions, corresponding to the basal levels of these immune parameters, and when the immune response was peaking, respectively [45, 71]. The immune challenge was performed by injecting of a 5µL suspension of the bacterium *Bacillus cereus* in Phosphate Buffer Saline (PBS 10 mM, pH 7.4), which was inactivated by fixation (see method below), immediately after the first hemolymph sample. Individuals were weighted before the first hemolymph sampling.

### Bacterial cultures for the immune challenge

The immune challenge involved the use of inactivated *Bacillus cereus* obtained from the Institut Pasteur (CIP69.12). The methods for bacterial culture and immune challenge were similar to those described by [63]. The bacteria were grown overnight in liquid broth medium (10 g bacto-tryptone, 5 g yeast extract, 10 g NaCl in 1,000 mL of distilled water, pH 7) at 28 °C. The bacteria were then killed in a 0.5% formaldehyde/PBS solution for 30 min, rinsed three time in PBS and their concentration adjusted to 10^8^cells/mL of PBS using a Neubauer improved cell counting chamber. The success of bacteria inactivation was confirmed by incubating of a sample of the inactivated bacterial solution on sterile Broth medium with 1% bacterial agar at 28 °C for 24 h. Aliquots of the inactivated bacterial solution were stored at -20 °C until their use for the immune challenge. During the Immune challenge, 5µL of the inactivate bacterial solution was injected into the bacterial region using a sterile glass capillary that has been shaped into a fine point using an electrode puller (Narashige PC-10).

### Hemolymph collection and immune measures

Insects were chilled on ice, and a small incision was made at the membrane between the thorax and the abdomen to collect 6µL of hemolymph, both before and 3 days after the immune challenge.

Out of the collected 6µL of hemolymph, 3 µL were mixed in 15µL of anticoagulant buffer (30 mM trisodium citrate, 26 mM citric acid, 15 mM sodium chloride, 20 mM EDTA, pH 4.6) [72]. Immediately, 10µL of this hemolymph-buffer mixture was used to estimate the concentrations of total hemocytes and different hemocyte types, using a Neubauer improved cell counter under a phase-contrast microscope (magnification × 400). The hemocyte types considered were granulocytes, plasmatocytes, prohemocytes and oenocitoids, which are the major hemocyte types in *T. molitor*. The identification of these hemocyte types was based on the description made by [26]. We did not measure the activity of the hemocytes, and these hemocyte counts will serve as a proxy for investment in the cellular response.

The remaining 3µL of hemolymph were mixed with 20µL PBS. These samples were reserved for measuring antibacterial activity and the enzymatic activity of the proPO system. For measuring antimicrobial activity only, a subsample of 5µL was transferred to a microcentrifuge tube coated with N-phenylthiourea to inhibit enzymatic activity. The remaining 18µL, standing in a tube without N-phenylthiourea, were used for proPO measurements. These samples were immediately flash-frozen in liquid nitrogen and stored at -80 °C.

Antibacterial activity was estimated using an inhibition zone assay, following the method described by Moret [73]. Plates were prepared with *Arthrobacter globiformis* (Pasteur Institute CIP105365) by adding an overnight culture of the bacterium to Broth medium with 1% bacterial agar, adjusted to a concentration of 10^6^cells/mL. Each plate contained 10 mL of the *A. globiformis*-seeded medium. During the solidification of the medium, 2 mm diameter wells were created using a rake. Hemolymph samples stored at -80 °C in N-phenylthiourea-coated microcentrifuge tubes were thawed on ice, and 2µL of each sample were placed in the experimental wells, and a positive control (2.5 mg/ml in absolute ethanol of Tetracycline: Sigma-Aldrich T3383) was included on each plate [66]. Presence or absence of an inhibition zone and its diameter were measured after 48 h of incubation at 28 °C. Due to slight variations in inhibition zones among plates resulting from tetracycline addition, the diameter of the zone of inhibition for each sample was adjusted to consider this variation between plates. To achieve this, the Petri dish with the largest inhibition zone from the tetracycline-positive control was used as a reference. Subsequently, for each sample, the inhibition zone was multiplied by the ratio of the inhibition zone caused by tetracycline in the reference Petri dish to that in the Petri dish where the sample was measured.

For each individual hemolymph sample, both the activity of naturally activated PO enzymes (PO activity) and the activity of proenzymes (proPO) in addition to PO (total-PO activity) were measured as a proxy of the proPO component of immunity, using a spectrophotometric assay [66]. The PO activity was quantified without further activation, while the total-PO activity required the activation of proPO into PO using chymotrypsin. For this purpose, frozen hemolymph samples were thawed on ice and then centrifuged (3,500 g, 5 min at 4 °C). Five µL of supernatant was added to a microplate well containing 20µL of PBS, and either 140µL of distilled water to measure PO activity only, or 140µL of chymotrypsin solution (Sigma-Aldrich C7762, 0.07 mg/mL of distilled water) to measure total-PO activity. Then, 20µL of L-Dopa solution (Sigma-Aldrich D9628, a 4 mg/mL of distilled water) was added to each well. The reaction was allowed to proceed at 30 °C in a microplate reader (Molecular Devices, SpectraMax iD3) for 40 min. Readings were taken every 36 s at 490 nm and analyzed using the SoftMax Pro 7.1.2. software (Molecular Devices). Enzyme activity was measured as the slope (Vmax value: change in absorbance unit per minute) of the reaction curve during the linear phase of the reaction and reported relative to the activity of 1µL of pure hemolymph.

### Statistical analysis

Statistical analyzes were performed with R version 4.1.2 with Rstudio 2022.12.0 [74].

Firstly, we verified that the changes in larval environments had indeed an impact on the growth duration and the adult longevity. As mass is a good indicator of individual quality, we also characterized the effect on the mass reached by individuals during their pupal phase. For these three measures (Growth duration, Adult longevity and Pupal mass), the effect of larval environment and sex was evaluated. The larval environmental temperature conditions were categorized as T20 (20.5 ± 1 °C and 85 ± 5% RH), T24 (24 ± 1 °C and 85 ± 5% RH) and T28 (28.5 ± 1 °C and 85 ± 5% RH). The relative humidity conditions were designated as H55 (55 ± 5% RH and 24 ± 1 °C), H70 (70 ± 5% RH and 24 ± 1 °C) and H85 (85 ± 5% RH and 24 ± 1 °C). It should be noted that conditions T24 and H85 refer to same group of insects. Generalized linear models with a gamma distribution and a log link function (package stats [74]) was used to assess the variation in pupal mass in both environmental conditions, and variation in growth duration under the different larval temperatures and sex. A linear model was used to analyze the effects of the different relative humidities and sex on growth duration. In all cases, estimated marginal means were calculated with the lsmeans function (package lsmeans [75]) for variables with a significant effect. For survival analysis, we employed Cox models (Surv function to establish life table and coxph, survival package [76]) to estimate the overall effects of larval environment and sex, followed by a Wald test. Gains or losses in survival chance between different modalities of the two factors were evaluated using a z-test.

As in [49], in order to obtain synthetic variables for the innate immune parameters of the individuals, Principal Component Analyses (PCA) were performed, using the PCA function from the FactoMineR package [77]. The variables included in the PCA were the concentrations of total hemocytes, granulocytes, plasmatocytes, prohemocytes, PO and total-PO activities, as well as the diameters of the inhibition zones from the antibacterial activity tests. For each of these variables, one measurement was taken before the immune challenge and one after. Prior to PCA, the variables were scaled. The resulting summarized scores of these immune parameters was then analyzed using linear mixed models (using the lmer function from the lme4 package [78]) with growth duration or adult longevity, immune challenge, age at which the immune challenge was conducted, and their two-way and three-way interactions as explanatory variables. Additionally, sex and body mass measured at the moment of the immune challenge were included in the models as main effects only. Larval environmental temperature and RH were included as a random effect depending on whether the growth duration and adult longevity were manipulated by the experimental modification of the larval temperature or RH. Since immune parameters were measured twice (before and after the immune challenge) for each individual insect, individual identity was also included as a random factor. Model selection on fixed effects was carried out by comparing the Akaike Information Criterion (AIC) of the exhaustive list of competing models. All models with a ΔAIC ≤ 2 relative to the model with the minimum AIC were considered as the best models [79]. Lists of the best models for each analysis are available in the supplementary material. From among these models, we selected those that included the most possible interactions, as these interactions precisely tested our hypotheses. The significance of the effects in the selected model was assessed using Wald tests (Anova function, car package [80]).

### Supplementary Information

**Additional file 1: Table S1.** Best models initially including Growth duration according to ΔAIC for Cellular immunity component (approached by the coordinates on the first principal component of an ACP) the individuals grown in the different temperature conditions (Larval environment: T20, T24 and T28). The worst model is given for information. The most completed model tested contained: the Growth duration, the Age at measurement (young: ~15 days of adult stage or older: ~45 days of adult age), Challenge (before or after the immune challenge), the mass before the immune challenge, two and three variables interactions between Growth duration, Age at measurement and Challenge. Models were linear mixed models with Larval environment and Individual as random effect. **Table S2.** Best models initially including Growth duration according to ΔAIC for Enzymatic immunity component (approached by the coordinates on the second principal component of an ACP) the individuals grown in the different temperature conditions (Larval environment: T20, T24 and T28). The worst model is given for information. The most completed model tested contained: the Growth duration, the Age at measurement (young: ~15 days of adult stage or older: ~45 days of adult age), Challenge (before or after the immune challenge), the mass before the immune challenge, two and three variables interactions between Growth duration, Age at measurement and Challenge. Models were linear mixed models with Larval environment and Individual as random effect. **Table S3.** Best models initially including Growth duration according to ΔAIC for Antibacterial activity component (approached by the coordinates on the third principal component of an ACP) the individuals grown in the different temperature conditions (Larval environment: T20, T24 and T28). The worst model is given for information. The most completed model tested contained: the Growth duration, the Age at measurement (young: ~15 days of adult stage or older: ~45 days of adult age), Challenge (before or after the immune challenge), the mass before the immune challenge, two and three variables interactions between Growth duration, Age at measurement and Challenge. Models were linear mixed models with Larval environment and Individual as random effect. **Table S4.** Best models initially including Adult longevity according to ΔAIC for Cellular immunity component (approached by the coordinates on the first principal component of an ACP) the individuals grown in the different temperature conditions (Larval environment: T20, T24 and T28). The worst model is given for information. The most completed model tested contained: the Adult longevity, the Age at measurement (young: ~15 days of adult stage or older: ~45 days of adult age), Challenge (before or after the immune challenge), the mass before the immune challenge, two and three variables interactions between Adult longevity, Age at measurement and Challenge. Models were linear mixed models with Larval environment and Individual as random effect. **Table S5.** Best models initially including Adult longevity according to ΔAIC for Enzymatic immunity component (approached by the coordinates on the second principal component of an ACP) the individuals grown in the different temperature conditions (Larval environment: T20, T24 and T28). The worst model is given for information. The most completed model tested contained: the Adult longevity, the Age at measurement (young: ~15 days of adult stage or older: ~45 days of adult age), Challenge (before or after the immune challenge), the mass before the immune challenge, two and three variables interactions between Adult longevity, Age at measurement and Challenge. Models were linear mixed models with Larval environment and Individual as random effect. **Table S6.** Best models initially including Adult longevity according to ΔAIC for Antibacterial activity component (approached by the coordinates on the third principal component of an ACP) the individuals grown in the different temperature conditions (Larval environment: T20, T24 and T28). The worst model is given for information. The most completed model tested contained: the Adult longevity, the Age at measurement (young: ~15 days of adult stage or older: ~45 days of adult age), Challenge (before or after the immune challenge), the mass before the immune challenge, two and three variables interactions between Adult longevity, Age at measurement and Challenge. Models were linear mixed models with Larval environment and Individual as random effect. **Table S7.** Best models initially including Growth duration according to ΔAIC for Cellular immunity component (approached by the coordinates on the first principal component of an ACP) the individuals grown in the different relative humidity conditions (Larval environment: H55, H70 and H85). The worst model is given for information. The most completed model tested contained: the Growth duration, the Age at measurement (young: ~15 days of adult stage or older: ~45 days of adult age), Challenge (before or after the immune challenge), the mass before the immune challenge, two and three variables interactions between Growth duration, Age at measurement and Challenge. Models were linear mixed models with Larval environment and Individual as random effect. **Table S8.** Best models initially including Growth duration according to ΔAIC for Enzymatic immunity component (approached by the coordinates on the second principal component of an ACP) the individuals grown in the different relative humidity conditions (Larval environment: H55, H70 and H85). The worst model is given for information. The most completed model tested contained: the Growth duration, the Age at measurement (young: ~15 days of adult stage or older: ~45 days of adult age), Challenge (before or after the immune challenge), the mass before the immune challenge, two and three variables interactions between Growth duration, Age at measurement and Challenge. Models were linear mixed models with Larval environment and Individual as random effect. **Table S9.** Best models initially including Growth duration according to ΔAIC for Antibacterial activity component (approached by the coordinates on the third principal component of an ACP) the individuals grown in the different relative humidity conditions (Larval environment: H55, H70 and H85). The worst model is given for information. The most completed model tested contained: the Growth duration, the Age at measurement (young: ~15 days of adult stage or older: ~45 days of adult age), Challenge (before or after the immune challenge), the mass before the immune challenge, two and three variables interactions between Growth duration, Age at measurement and Challenge. Models were linear mixed models with Larval environment and Individual as random effect. **Table S10.** Best models initially including Adult longevity according to ΔAIC for Cellular immunity component (approached by the coordinates on the first principal component of an ACP) the individuals grown in the different relative humidity conditions (Larval environment: H55, H70 and H85). The worst model is given for information. The most completed model tested contained: the Adult longevity, the Age at measurement (young: ~15 days of adult stage or older: ~45 days of adult age), Challenge (before or after the immune challenge), the mass before the immune challenge, two and three variables interactions between Adult longevity, Age at measurement and Challenge. Models were linear mixed models with Larval environment and Individual as random effect. **Table S11.** Best models initially including Adult longevity according to ΔAIC for Enzymatic immunity component (approached by the coordinates on the second principal component of an ACP) the individuals grown in the different relative humidity conditions (Larval environment: H55, H70 and H85). The worst model is given for information. The most completed model tested contained: the Adult longevity, the Age at measurement (young: ~15 days of adult stage or older: ~45 days of adult age), Challenge (before or after the immune challenge), the mass before the immune challenge, two and three variables interactions between Adult longevity, Age at measurement and Challenge. Models were linear mixed models with Larval environment and Individual as random effect. **Table S12.** Best models initially including Adult longevity according to ΔAIC for Antibacterial activity component (approached by the coordinates on the third principal component of an ACP) the individuals grown in the different relative humidity conditions (Larval environment: H55, H70 and H85). The worst model is given for information. The most completed model tested contained: the Adult longevity, the Age at measurement (young: ~15 days of adult stage or older: ~45 days of adult age), Challenge (before or after the immune challenge), the mass before the immune challenge, two and three variables interactions between Adult longevity, Age at measurement and Challenge. Models were linear mixed models with Larval environment and Individual as random effect.

## Data Availability

The datasets used and/or analyzed during the current study are available from the corresponding author on reasonable request.
