# Supplementary material for: Growth and longevity modulation through larval environment mediate immunosenescence and immune strategy of Tenebrio molitor
Source: Immun Ageing. 2024 Jan 12;21:7. doi: 10.1186/s12979-023-00409-w (PMC10785379; doi:10.1186/s12979-023-00409-w)
Supplement: Supplementary file 1 — Additional file 1: Table S1. Best models initially including Growth duration according to ΔAIC for Cellular immunity component (approached by the coordinates on the first principal component of an ACP) the individuals grown in the different temperature conditions (Larval environment: T20, T24 and T28). The worst model is given for information. The most completed model tested contained: the Growth duration, the Age at measurement (young: ~15 days of adult stage or older: ~45 days of adult age), Challenge (before or after the immune challenge), the mass before the immune challenge, two and three variables interactions between Growth duration, Age at measurement and Challenge. Models were linear mixed models with Larval environment and Individual as random effect. Table S2. Best models initially including Growth duration according to ΔAIC for Enzymatic immunity component (approached by the coordinates on the second principal component of an ACP) the individuals grown in the different temperature conditions (Larval environment: T20, T24 and T28). The worst model is given for information. The most completed model tested contained: the Growth duration, the Age at measurement (young: ~15 days of adult stage or older: ~45 days of adult age), Challenge (before or after the immune challenge), the mass before the immune challenge, two and three variables interactions between Growth duration, Age at measurement and Challenge. Models were linear mixed models with Larval environment and Individual as random effect. Table S3. Best models initially including Growth duration according to ΔAIC for Antibacterial activity component (approached by the coordinates on the third principal component of an ACP) the individuals grown in the different temperature conditions (Larval environment: T20, T24 and T28). The worst model is given for information. The most completed model tested contained: the Growth duration, the Age at measurement (young: ~15 days of adult stage or older: ~45 days of [file 12979_2023_409_MOESM1_ESM.pdf]

Table S1: Best models initially including Growth duration according to ΔAIC for Cellular immunity component (approached by the coordinates on the first principal component of an ACP) the individuals grown in the different temperature conditions (Larval environment: T20, T24 and T28). The worst model is given for information. The most completed model tested contained: the Growth duration, the Age at measurement (young: ~15 days of adult stage or older: ~45 days of adult age), Challenge (before or after the immune challenge), the mass before the immune challenge, two and three variables interactions between Growth duration, Age at measurement and Challenge. Models were linear mixed models with Larval environment and Individual as random effect.

Models	AIC	ΔAIC
PC1 ~ Growth duration + Mass before challenge + Challenge + Age at measurement + Age at measurement : Challenge + Growth duration : Age at measurement : Challenge + (1|Individual) + (1|Larval environment)	2044.55	0.00
PC1 ~ Growth duration + Mass before challenge + Challenge + Age at measurement + Growth duration : Age at measurement + Growth duration : Age at measurement : Challenge + (1|Individual) + (1|Larval environment)	2044.67	0.11
**PC1 ~ Growth duration + Mass before challenge + Challenge + Age at measurement + Growth duration : Challenge + Growth duration : Age at measurement : Challenge + (1|Individual) + (1|Larval environment)**	**2044.67**	**0.11**
PC1 ~ Growth duration + Mass before challenge + Challenge + Age at measurement + Growth duration : Age at measurement + (1|Individual) + (1|Larval environment)	2045.90	1.35
PC1 ~ Growth duration + Mass before challenge + Challenge + Age at measurement + Sex + Age at measurement : Challenge + Growth duration : Age at measurement : Challenge + (1|Individual) + (1|Larval environment)	2046.54	1.99
…		
PC1 ~ Sex + (1|Individual) + (1|Larval environment)	2089.15	44.59

Table S2: Best models initially including Growth duration according to ΔAIC for Enzymatic immunity component (approached by the coordinates on the second principal component of an ACP) the individuals grown in the different temperature conditions (Larval environment: T20, T24 and T28). The worst model is given for information. The most completed model tested contained: the Growth duration, the Age at measurement (young: ~15 days of adult stage or older: ~45 days of adult age), Challenge (before or after the immune challenge), the mass before the immune challenge, two and three variables interactions between Growth duration, Age at measurement and Challenge. Models were linear mixed models with Larval environment and Individual as random effect.

Models	AIC	ΔAIC
**PC2 ~ Challenge + Mass before challenge + Growth duration + Growth duration : Challenge + (1|Individual) + (1|Larval environment)**	**1714.45**	**0.00**
PC2 ~ Challenge + Mass before challenge + Age at measurement + Growth duration + Growth duration : Challenge + (1|Individual) + (1|Larval environment)	1714.71	0.26
PC2 ~ Challenge + Growth duration + Growth duration : Challenge + (1|Individual) + (1|Larval environment)	1715.14	0.69

PC2 ~ Challenge + Mass before challenge + (1|Individual) + (1|Larval environment)	1715.77	1.32
PC2 ~ Challenge + Mass before challenge + Growth duration + Sex + Growth duration : Challenge + (1|Individual) + (1|Larval environment)	1715.88	1.43
PC2 ~ Challenge + Age at measurement + Growth duration + Growth duration : Challenge + (1|Individual) + (1|Larval environment)	1715.91	1.46
PC2 ~ Challenge + Mass before challenge + Age at measurement + (1|Individual) + (1|Larval environment)	1716.10	1.65
PC2 ~ Challenge + Mass before challenge + Age at measurement + Growth duration + Sex + Growth duration : Challenge + (1|Individual) + (1|Larval environment)	1716.21	1.76
…		
PC2 ~ Age at measurement + Growth duration + Sex + Growth duration : Age at measurement + (1|Individual) + (1|Larval environment)	1773.89	59.44

Table S3: Best models initially including Growth duration according to ΔAIC for Antibacterial activity component (approached by the coordinates on the third principal component of an ACP) the individuals grown in the different temperature conditions (Larval environment: T20, T24 and T28). The worst model is given for information. The most completed model tested contained: the Growth duration, the Age at measurement (young: ~15 days of adult stage or older: ~45 days of adult age), Challenge (before or after the immune challenge), the mass before the immune challenge, two and three variables interactions between Growth duration, Age at measurement and Challenge. Models were linear mixed models with Larval environment and Individual as random effect.

Models	AIC	ΔAIC
**PC3 ~ Challenge + Age at measurement + Growth duration + Age at measurement : Challenge + Growth duration : Age at measurement : Challenge + (1|Individual) + (1|Larval environment)**	**981.61**	**0.00**
PC3 ~ Challenge + Mass before challenge + Age at measurement + Growth duration + Age at measurement : Challenge + Growth duration : Age at measurement : Challenge + (1|Individual) + (1|Larval environment)	983.29	1.68
PC3 ~ Challenge + Age at measurement + Growth duration + Sex + Age at measurement : Challenge + Growth duration : Age at measurement : Challenge + (1|Individual) + (1|Larval environment)	983.57	1.96
…		
PC3 ~ Growth duration + Sex + (1|Individual) + (1|Larval environment)	1591.00	609.39

Table S4: Best models initially including Adult longevity according to ΔAIC for Cellular immunity component (approached by the coordinates on the first principal component of an ACP) the individuals grown in the different temperature conditions (Larval environment: T20, T24 and T28). The worst model is given for information. The most

completed model tested contained: the Adult longevity, the Age at measurement (young: ~15 days of adult stage or older: ~45 days of adult age), Challenge (before or after the immune challenge), the mass before the immune challenge, two and three variables interactions between Adult longevity, Age at measurement and Challenge. Models were linear mixed models with Larval environment and Individual as random effect.

Models	AIC	ΔAIC
PC1 ~ Mass before challenge + Challenge + Adult longevity + (1|Individual) + (1|Larval environment)	2053.93	0.00
PC1 ~ Mass before challenge + Challenge + Age at measurement + Adult longevity + (1|Individual) + (1|Larval environment)	2054.10	0.17
**PC1 ~ Mass before challenge + Challenge + Age at measurement + Adult longevity + Age at measurement : Challenge + (1|Individual) + (1|Larval environment)**	**2054.96**	**1.03**
PC1 ~ Mass before challenge + Challenge + Adult longevity + Adult longevity : Challenge + (1|Individual) + (1|Larval environment)	2055.58	1.65
PC1 ~ Mass before challenge + Challenge + Age at measurement + Adult longevity + Adult longevity : Age at measurement + (1|Individual) + (1|Larval environment)	2055.68	1.75
PC1 ~ Mass before challenge + Challenge + Age at measurement + Adult longevity + Adult longevity : Challenge + (1|Individual) + (1|Larval environment)	2055.75	1.82
PC1 ~ Mass before challenge + Challenge + Adult longevity + Sex + (1|Individual) + (1|Larval environment)	2055.89	1.96
…		
PC1 ~ Sex + (1|Individual) + (1|Larval environment)	2089.15	35.22

Table S5: Best models initially including Adult longevity according to ΔAIC for Enzymatic immunity component (approached by the coordinates on the second principal component of an ACP) the individuals grown in the different temperature conditions (Larval environment: T20, T24 and T28). The worst model is given for information. The most completed model tested contained: the Adult longevity, the Age at measurement (young: ~15 days of adult stage or older: ~45 days of adult age), Challenge (before or after the immune challenge), the mass before the immune challenge, two and three variables interactions between Adult longevity, Age at measurement and Challenge. Models were linear mixed models with Larval environment and Individual as random effect.

Models	AIC	ΔAIC
**PC2 ~ Challenge + Mass before challenge + Adult longevity + Adult longevity : Challenge + (1|Individual) + (1|Larval environment)**	**1707.03**	**0.00**
PC2 ~ Challenge + Mass before challenge + Adult longevity + (1|Individual) + (1|Larval environment)	1708.16	1.13

PC2 ~ Challenge + Mass before challenge + Adult longevity + Age at measurement + Adult longevity : Challenge + (1|Individual) + (1|Larval environment)	1708.21	1.19
PC2 ~ Challenge + Mass before challenge + Adult longevity + Sex + Adult longevity : Challenge + (1|Individual) + (1|Larval environment)	1708.31	1.28
…		
PC2 ~ Age at measurement + Sex + (1|Individual) + (1|Larval environment)	1771.60	64.57

Table S6: Best models initially including Adult longevity according to ΔAIC for Antibacterial activity component (approached by the coordinates on the third principal component of an ACP) the individuals grown in the different temperature conditions (Larval environment: T20, T24 and T28). The worst model is given for information. The most completed model tested contained: the Adult longevity, the Age at measurement (young: ~15 days of adult stage or older: ~45 days of adult age), Challenge (before or after the immune challenge), the mass before the immune challenge, two and three variables interactions between Adult longevity, Age at measurement and Challenge. Models were linear mixed models with Larval environment and Individual as random effect.

Models	AIC	ΔAIC
PC3 ~ Challenge + Mass before challenge + Age at measurement + Adult longevity + Age at measurement : Challenge + Adult longevity : Age at measurement : Challenge + (1|Individual) + (1|Larval environment)	995.28	0.00
**PC3 ~ Challenge + Age at measurement + Adult longevity + Age at measurement : Challenge + Adult longevity : Age at measurement : Challenge + (1|Individual) + (1|Larval environment)**	**995.32**	**0.04**
PC3 ~ Challenge + Age at measurement + Adult longevity + Age at measurement : Challenge + (1|Individual) + (1|Larval environment)	996.14	0.86
PC3 ~ Challenge + Mass before challenge + Age at measurement + Adult longevity + Age at measurement : Challenge + (1|Individual) + (1|Larval environment)	996.43	1.15
PC3 ~ Challenge + Mass before challenge + Age at measurement + Adult longevity + Adult longevity : Challenge + Adult longevity : Age at measurement : Challenge + (1|Individual) + (1|Larval environment)	996.52	1.24
PC3 ~ Challenge + Mass before challenge + Age at measurement + Adult longevity + Adult longevity : Age at measurement + Adult longevity : Age at measurement : Challenge + (1|Individual) + (1|Larval environment)	996.52	1.24
PC3 ~ Challenge + Age at measurement + Adult longevity + Adult longevity : Age at measurement + Adult longevity : Age at measurement : Challenge + (1|Individual) + (1|Larval environment)	996.56	1.28
PC3 ~ Challenge + Age at measurement + Adult longevity + Adult longevity : Challenge + Adult longevity : Age at measurement : Challenge + (1|Individual) + (1|Larval environment)	996.56	1.28
PC3 ~ Challenge + Age at measurement + Adult longevity + Sex + Age at measurement : Challenge + Adult longevity : Age at measurement : Challenge + (1|Individual) + (1|Larval environment)	997.25	1.97

PC3 ~ Challenge + Mass before challenge + Age at measurement + Adult longevity + Sex + Age at measurement : Challenge + Adult longevity : Age at measurement : Challenge + (1|Individual) + (1|Larval environment)	997.28	2.00
…		
PC3 ~ Sex + (1|Individual) + (1|Larval environment)	1590.90	595.62

Table S7: Best models initially including Growth duration according to ΔAIC for Cellular immunity component (approached by the coordinates on the first principal component of an ACP) the individuals grown in the different relative humidity conditions (Larval environment: H55, H70 and H85). The worst model is given for information. The most completed model tested contained: the Growth duration, the Age at measurement (young: ~15 days of adult stage or older: ~45 days of adult age), Challenge (before or after the immune challenge), the mass before the immune challenge, two and three variables interactions between Growth duration, Age at measurement and Challenge. Models were linear mixed models with Larval environment and Individual as random effect.

Models	AIC	ΔAIC
PC1 ~ Challenge + Mass before challenge + Sex + Growth duration + (1|Individual) + (1|Larval environment)	1912.18	0.00
PC1 ~ Challenge + Mass before challenge + Sex + Growth duration + Age at measurement + (1|Individual) + (1|Larval environment)	1913.35	1.17
PC1 ~ Challenge + Mass before challenge + Growth duration + (1|Individual) + (1|Larval environment)	1913.74	1.56
**PC1 ~ Challenge + Mass before challenge + Sex + Growth duration + Growth duration : Challenge + (1|Individual) + (1|Larval environment)**	**1913.98**	**1.81**
…		
PC1 ~ Age at measurement + (1|Individual) + (1|Larval environment)	1941.85	29.67

Table S8: Best models initially including Growth duration according to ΔAIC for Enzymatic immunity component (approached by the coordinates on the second principal component of an ACP) the individuals grown in the different relative humidity conditions (Larval environment: H55, H70 and H85). The worst model is given for information. The most completed model tested contained: the Growth duration, the Age at measurement (young: ~15 days of adult stage or older: ~45 days of adult age), Challenge (before or after the immune challenge), the mass before the immune challenge, two and three variables interactions between Growth duration, Age at measurement and Challenge. Models were linear mixed models with Larval environment and Individual as random effect.

Models	AIC	ΔAIC

**PC2 ~ Challenge + Growth duration + Age at measurement + Age at measurement : Challenge + (1|Individual) + (1|Larval environment)**	**1634.71**	**0.00**
PC2 ~ Challenge + Mass before challenge + Growth duration + Age at measurement + Age at measurement : Challenge + (1|Individual) + (1|Larval environment)	1635.41	0.70
PC2 ~ Challenge + Growth duration + Age at measurement + Sex + Age at measurement : Challenge + (1|Individual) + (1|Larval environment)	1635.46	0.75
PC2 ~ Challenge + Mass before challenge + Growth duration + Age at measurement + Sex + Age at measurement : Challenge + (1|Individual) + (1|Larval environment)	1635.79	1.08
PC2 ~ Challenge + Growth duration + (1|Individual) + (1|Larval environment)	1636.07	1.36
PC2 ~ Challenge + Growth duration + Age at measurement + (1|Individual) + (1|Larval environment)	1636.33	1.62
…		
PC2 ~ Age at measurement + Sex + (1|Individual) + (1|Larval environment)	1681.87	47.15

Table S9: Best models initially including Growth duration according to ΔAIC for Antibacterial activity component (approached by the coordinates on the third principal component of an ACP) the individuals grown in the different relative humidity conditions (Larval environment: H55, H70 and H85). The worst model is given for information. The most completed model tested contained: the Growth duration, the Age at measurement (young: ~15 days of adult stage or older: ~45 days of adult age), Challenge (before or after the immune challenge), the mass before the immune challenge, two and three variables interactions between Growth duration, Age at measurement and Challenge. Models were linear mixed models with Larval environment and Individual as random effect.

Models	AIC	ΔAIC
PC3 ~ Challenge + Age at measurement + Mass before challenge + Growth duration + Growth duration : Challenge + Growth duration : Age at measurement : Challenge + (1|Individual) + (1|Larval environment)	1039.22	0.00
**PC3 ~ Challenge + Age at measurement + Mass before challenge + Growth duration + Growth duration : Age at measurement + Growth duration : Age at measurement : Challenge + (1|Individual) + (1|Larval environment)**	**1039.22**	**0.00**
PC3 ~ Challenge + Age at measurement + Mass before challenge + Growth duration + Growth duration : Challenge + (1|Individual) + (1|Larval environment)	1039.31	0.09
PC3 ~ Challenge + Age at measurement + Growth duration + Growth duration : Age at measurement + Growth duration : Age at measurement : Challenge + (1|Individual) + (1|Larval environment)	1039.37	0.14
PC3 ~ Challenge + Age at measurement + Growth duration + Growth duration : Challenge + Growth duration : Age at measurement : Challenge + (1|Individual) + (1|Larval environment)	1039.37	0.14

PC3 ~ Challenge + Age at measurement + Growth duration + Growth duration : Challenge + (1|Individual) + (1|Larval environment)	1039.57	0.35
PC3 ~ Challenge + Age at measurement + Mass before challenge + Growth duration + Age at measurement : Challenge + (1|Individual) + (1|Larval environment)	1040.86	1.64
PC3 ~ Challenge + Age at measurement + Mass before challenge + Growth duration + (1|Individual) + (1|Larval environment)	1040.90	1.67
PC3 ~ Challenge + Age at measurement + Growth duration + Age at measurement : Challenge + (1|Individual) + (1|Larval environment)	1041.14	1.91
PC3 ~ Challenge + Age at measurement + Growth duration + (1|Individual) + (1|Larval environment)	1041.19	1.96
PC3 ~ Challenge + Age at measurement + Mass before challenge + Growth duration + Age at measurement : Challenge + Growth duration : Age at measurement : Challenge + (1|Individual) + (1|Larval environment)	1041.22	1.99
PC3 ~ Challenge + Age at measurement + Mass before challenge + Growth duration + Sex + Growth duration : Age at measurement + Growth duration : Age at measurement : Challenge + (1|Individual) + (1|Larval environment)	1041.22	2.00
PC3 ~ Challenge + Age at measurement + Mass before challenge + Growth duration + Sex + Growth duration : Challenge + Growth duration : Age at measurement : Challenge + (1|Individual) + (1|Larval environment)	1041.22	2.00
…		
PC3 ~ Sex + (1|Individual) + (1|Larval environment)	1488.32	449.10

Table S10: Best models initially including Adult longevity according to ΔAIC for Cellular immunity component (approached by the coordinates on the first principal component of an ACP) the individuals grown in the different relative humidity conditions (Larval environment: H55, H70 and H85). The worst model is given for information. The most completed model tested contained: the Adult longevity, the Age at measurement (young: ~15 days of adult stage or older: ~45 days of adult age), Challenge (before or after the immune challenge), the mass before the immune challenge, two and three variables interactions between Adult longevity, Age at measurement and Challenge. Models were linear mixed models with Larval environment and Individual as random effect.

Models	AIC	ΔAIC
PC1 ~ Challenge + Mass before challenge + Sex + (1|Individual) + (1|Larval environment)	1924.65	0.00
**PC1 ~ Challenge + Mass before challenge + Sex + Adult longevity + Adult longevity : Challenge + (1|Individual) + (1|Larval environment)**	**1925.47**	**0.82**
PC1 ~ Challenge + Mass before challenge + Sex + Adult longevity + (1|Individual) + (1|Larval environment)	1925.83	1.18

PC1 ~ Challenge + Mass before challenge + Sex + Age at measurement + (1|Individual) + (1|Larval environment)	1926.04	1.39
PC1 ~ Challenge + Mass before challenge + (1|Individual) + (1|Larval environment)	1926.46	1.81
…		
PC1 ~ Age at measurement + Adult longevity + Adult longevity : Age at measurement + (1|Individual) + (1|Larval environment)	1953.90	29.25

Table S11: Best models initially including Adult longevity according to ΔAIC for Enzymatic immunity component (approached by the coordinates on the second principal component of an ACP) the individuals grown in the different relative humidity conditions (Larval environment: H55, H70 and H85). The worst model is given for information. The most completed model tested contained: the Adult longevity, the Age at measurement (young: ~15 days of adult stage or older: ~45 days of adult age), Challenge (before or after the immune challenge), the mass before the immune challenge, two and three variables interactions between Adult longevity, Age at measurement and Challenge. Models were linear mixed models with Larval environment and Individual as random effect.

Models	AIC	ΔAIC
**PC2 ~ Challenge + Age at measurement + Age at measurement : Challenge + (1|Individual) + (1|Larval environment)**	**1649.36**	**0.00**
PC2 ~ Challenge + Sex + Age at measurement + Age at measurement : Challenge + (1|Individual) + (1|Larval environment)	1649.79	0.43
PC2 ~ Challenge + (1|Individual) + (1|Larval environment)	1650.20	0.84
PC2 ~ Challenge + Mass before challenge + Age at measurement + Age at measurement : Challenge + (1|Individual) + (1|Larval environment)	1650.29	0.93
PC2 ~ Challenge + Mass before challenge + Sex + Age at measurement + Age at measurement : Challenge + (1|Individual) + (1|Larval environment)	1650.33	0.97
PC2 ~ Challenge + Sex + (1|Individual) + (1|Larval environment)	1650.62	1.26
PC2 ~ Challenge + Age at measurement + (1|Individual) + (1|Larval environment)	1651.08	1.72
PC2 ~ Challenge + Age at measurement + Adult longevity + Age at measurement : Challenge + (1|Individual) + (1|Larval environment)	1651.35	1.99
PC2 ~ Challenge + Mass before challenge + (1|Individual) + (1|Larval environment)	1651.36	2.00

…		
PC2 ~ Sex + Age at measurement + Adult longevity + Adult longevity : Age at measurement + (1|Individual) + (1|Larval environment)	1694.25	44.89

Table S12: Best models initially including Adult longevity according to ΔAIC for Antibacterial activity component (approached by the coordinates on the third principal component of an ACP) the individuals grown in the different relative humidity conditions (Larval environment: H55, H70 and H85). The worst model is given for information. The most completed model tested contained: the Adult longevity, the Age at measurement (young: ~15 days of adult stage or older: ~45 days of adult age), Challenge (before or after the immune challenge), the mass before the immune challenge, two and three variables interactions between Adult longevity, Age at measurement and Challenge. Models were linear mixed models with Larval environment and Individual as random effect.

Models	AIC	ΔAIC
**PC3 ~ Challenge + Age at measurement + Adult longevity + Age at measurement : Challenge + Adult longevity : Age at measurement : Challenge + (1|Individual) + (1|Larval environment)**	**1061.44**	**0.00**
PC3 ~ Challenge + Age at measurement + Mass before challenge + Adult longevity + Age at measurement : Challenge + Adult longevity : Age at measurement : Challenge + (1|Individual) + (1|Larval environment)	1061.78	0.34
PC3 ~ Challenge + Age at measurement + Adult longevity + Sex + Age at measurement : Challenge + Adult longevity : Age at measurement : Challenge + (1|Individual) + (1|Larval environment)	1062.78	1.34
PC3 ~ Challenge + Age at measurement + Adult longevity + Adult longevity : Challenge + (1|Individual) + (1|Larval environment)	1062.88	1.45
PC3 ~ Challenge + Age at measurement + Mass before challenge + Adult longevity + Sex + Age at measurement : Challenge + Adult longevity : Age at measurement : Challenge + (1|Individual) + (1|Larval environment)	1063.36	1.92
PC3 ~ Challenge + Age at measurement + Mass before challenge + Adult longevity + Adult longevity : Challenge + (1|Individual) + (1|Larval environment)	1063.42	1.98
…		
PC3 ~ Adult longevity + Sex + (1|Individual) + (1|Larval environment)	1498.63	437.19
